# Patterns of Reproductive Management in Sheep and Goat Farms in Greece

**DOI:** 10.3390/ani12243455

**Published:** 2022-12-07

**Authors:** Daphne T. Lianou, Natalia G. C. Vasileiou, Charalambia K. Michael, Irene Valasi, Vasia S. Mavrogianni, Mariangela Caroprese, George C. Fthenakis

**Affiliations:** 1 Veterinary Faculty, University of Thessaly, 43100 Karditsa, Greece; 2 Faculty of Animal Science, University of Thessaly, 41110 Larissa, Greece; 3 Department of Agriculture, Food, Natural Resources and Engineering (DAFNE), University of Foggia, 71122 Foggia, Italy

**Keywords:** goat, lambing, pregnancy, pregnancy diagnosis, reproduction control, reproduction pattern, reproductive management, seasonality, sheep, vaccination

## Abstract

**Simple Summary:**

The paper presents practices related to reproductive management in sheep and goat farms in Greece. It presents descriptive information regarding the practices followed and the predictors associated with them. The results also indicate that various reproductive practices can have significant effects on the production outcomes (milk production, lamb production) in sheep and goat farms. The correct implementation of reproductive management in sheep and goat farms, based on scientific principles and compliance with established regulations, is important for the improvement in the welfare of farm animals and high production outcomes.

**Abstract:**

This paper reports findings regarding patterns of reproductive management in 325 sheep flocks and 119 goat herds throughout Greece. The objectives were to describe the patterns of reproductive management in sheep and goat farms, to highlight factors that were associated with these management patterns and the clinical application of the various reproductive management approaches in the farms and to establish potential associations with production outcomes. The median months of the start of the mating period for adult sheep and goats were May and June, respectively and for ewe lambs and replacement goats these were August and September, respectively. The median duration of the mating period was 2 months for adults and 1 month for replacements. The median ratio of female to male animals was 22:1 and 25:1 in sheep and goat farms, respectively. Control of reproduction was applied in 33.2% and 16.8% of sheep and goat farms, respectively, mostly by intravaginal progestagen sponges and less often by melatonin implants; multivariable analysis indicated five (availability of milking parlour, number of ewes in a farm, number of daily milkings, daily period spent by farmers at the farm, farming tradition in the family) and two (number of daily milkings, availability of milking parlour) predictors in sheep and goat farms, respectively. Pregnancy diagnosis ultrasonographically was performed in 36.6% and 16.8% of sheep and goat farms, respectively; multivariable analysis indicated as significant three (management system applied in farms, age of farmer, farming tradition in the family) and two (management system applied in farms, availability of milking parlour) predictors in sheep and goat farms, respectively. The control of reproduction and pregnancy diagnosis were associated with a higher number of vaccinations during pregnancy: 2.6 and 2.7, respectively, versus 2.2 and 2.1 in farms where they were not performed. The average per farm number of lambs/kids born per female animal was 1.33 ± 0.01 and 1.30 ± 0.02 for sheep and goat farms, respectively; five (control of reproduction, location of the farm, presence of working staff, collaboration with veterinary practice, breed of ewes) and two (control of reproduction, breed of female goats) predictors were identified in sheep and goat farms, respectively, for high numbers of lambs/kids. Lambs/kids were taken away from their dams at the age of 50 and 65 days, respectively. The average culling age (females, males) was 5.9 and 4.4 years and 6.9 and 4.9 years for sheep and goats, respectively. Farmers sourced animals to be used as replacements for those removed from the farm, mostly from the animals in their own farms, considering criteria related to milk production. Finally, the application of the control of reproduction was associated with increased milk production and the number of newborns, whilst an inverse correlation between the culling age of animals and milk production was seen.

## 1. Introduction

In the context of health management in sheep and goat farms, the evaluation and recording of patterns of reproductive activity is important, as it is integrally linked with the production outcomes in the flocks and herds. In dairy sheep and goats, the reproductive cycle often coincides with the annual production cycle: mating, pregnancy, parturition, weaning of newborns (at various ages), milking and so forth. There is interest in the study of reproductive patterns applied in the farms, as it can provide information in relation to typology and the general management of these farms.

Internationally, there is little information in the description of patterns of reproductive management applied in sheep and goat farms. For the identification of relevant articles, we initiated a search of the relevant literature in the Web of Science database by using the search terms ‘[*reproductive management*] AND [*sheep* OR goat*]’. Irrelevant records (e.g., referring to other animal species) were then excluded and the remaining articles were assessed individually. Thus, 39 articles were considered to cover fully and precisely the intended search. Of these, 27 (69%) were published in 2019 and thereafter, which indicates the current increased interest in this topic. Among those recently published studies, Rivas et al. [1] presented the information collected from 157 sheep farms in Spain, by means of a structured questionnaire with 150 questions related to reproduction and breeding indicators in the flocks; Robertson et al. [2] reported an online survey, with 43 respondents, to assess reproductive management practices and their potential interactions within sheep flocks in Australia. With regard to goat farms, Alemayehu et al. [3] surveyed 242 goat farms in Ethiopia and recorded the reproductive patterns, with the aim to assess the management practices performed in that country, whilst Niang et al. [4] recorded the reproductive management details of 3138 female goats in India and reported the relevant reproductive performance outcomes.

Dairy sheep and goat farming is an important sector of the agricultural industry in Greece, with significant annual milk production. Greece is unique among European countries as the only country where milk production from sheep and goats exceeds that from cattle. In fact, total milk quantities from sheep and goats amount, respectively, to around 645,000 and 143,000 m^3^ annually [5]. Overall, these quantities cover approximately 15% of total European milk production from sheep and goats [6], thus confirming Greece as an important producer of sheep and goat milk in the continent.

Despite the importance of sheep and goat farming for the food production sector in Greece, the patterns of reproductive management in the farms have not been described. There is nevertheless scope for their monitoring, given that reproductive performance is an important determinant of production outcomes in the farms.

This paper reports findings regarding the patterns of reproductive management of small ruminants (specifically sheep and goats), as found during an extensive countrywide investigation carried out in 325 sheep flocks and 119 goat herds throughout Greece. Farms in all of the 13 administrative regions of the country were included in the study. The objectives of the study were (a) to describe the patterns of reproductive management in sheep and goat farms, (b) to highlight factors that were associated with these management patterns and the clinical application of the various reproductive management approaches in the farms and (c) to establish potential associations with production outcomes.

## 2. Materials and Methods

### 2.1. Sheep and Goat Farms and Collection of Information

**Figure 1:**
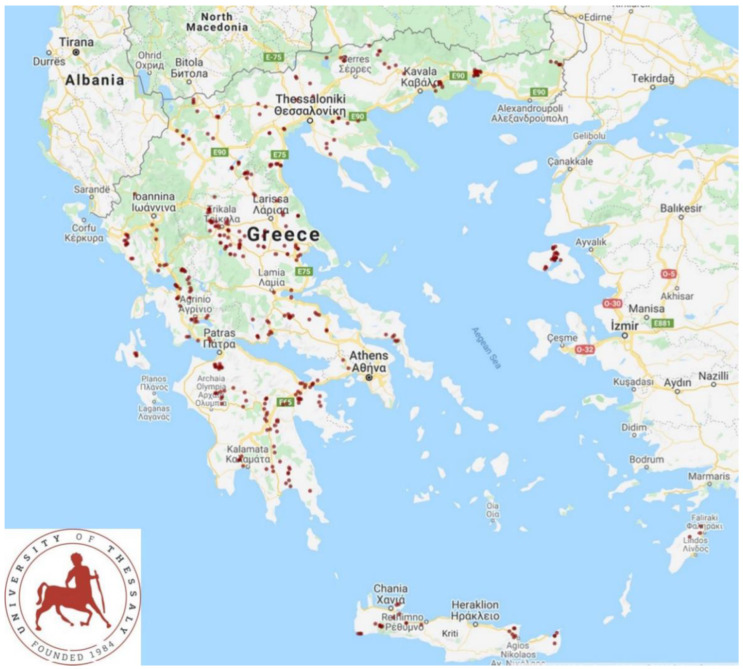
Location of the 444 small ruminant farms around Greece, which were visited to record details on patterns of reproductive management.

This study was carried out from April 2019 to July 2020 and involved a total of 325 sheep flocks and 119 goat herds (Figure 1). The farms entered into the study on a convenience basis (willingness of shepherds and goatherds to accept a visit by university personnel for interview and sample collection), as detailed previously [7,8]. The investigators visited all the farms personally for collection of information and samples.

Veterinarians active in small ruminant health management around Greece were contacted by telephone and asked if they wished to collaborate in the investigation [7]. A total of 47 veterinarians collaborated in this study. Initially, the veterinarian accompanying the investigators to the respective farm introduced them to the farmer. The senior investigator (author G.C.F.) then explained to the farmer the objectives and the details of the study and also introduced to the farmer the person that carried out the interview (author D.T.L.).

The interview was performed using a structured detailed questionnaire. Before the start of the main study, the questionnaire used to collect data from the farmers was tested for content validity [7]. The interview included general questions, as well as questions regarding infrastructure, animals, production characteristics, health management and human resources on the farm [7]. If farmers asked for clarification of the questions during the interview, these were provided immediately by the interviewer.

During the visits in the farms, samples of bulk tank milk were collected for somatic cell counts, total bacterial counts and milk composition measurement. Full details of the collection and the processing of samples have been presented before [9].

### 2.2. Data Management and Analysis

Data were entered into Microsoft Excel. Initially, we carried out basic descriptive analyses, and we obtained exact binomial confidence intervals (CIs). Results in sheep and goat farms were analysed and presented separately.

Data on farm location were collected in the field using hand-held Global Positioning System units; the geo-references were resolved to specific farm level. ArcGIS software (ESRI; Redlands, CA, USA) was employed for description and analysis of spatial information. Then, farms were classified in three areas, specifically north, central, south, by allocating them according to the latitude of their location.

Details on vaccinations during pregnancy against four infections, specifically, clostridial infections, contagious agalactia, pneumonia, staphylococcal mastitis, were obtained from previous work [8].

For the relevant statistical analyses, somatic cell counts (SCC) were transformed to somatic cell scores (SCS) as described by Wiggans and Shook [10] and Franzoi et al. [11]: SCS = log_2_(SCC/100) + 3, whilst total bacterial counts were transformed to log_10_ and the transformed data were used in the analyses. Then, for presentation of the results, the transformed findings were back-transformed into 100 × 2^(SCS − 3)^ and 10^log^ data, respectively.

Comparisons were performed using Pearson’s chi-squared test or analysis of variance or analysis of correlation, as appropriate.

The following outcomes were considered: ‘application of reproductive control’, ‘pregnancy diagnosis by means of ultrasonographic examination’ and ‘high number of lambs/kids born per ewe/female goat’ (for this analysis, ‘high number’ was deemed to be above the average of all the sheep/goat farms in the study). Variables evaluated for potential associations with the above outcomes are in Table S1; we obtained outcomes for these variables directly, i.e., from the answers of farmers during the interview performed at the farm, or, alternatively, we calculated outcomes based on the answers of the farmers. For each of these variables, categories were created according to the answers of the farmers. Separate analyses were performed for sheep and for goat farms. Exact binomial CIs were obtained. Initially, for each of the above right outcomes, univariable analysis was performed in cross-tabulation and with simple logistic regression. Subsequently, again separately for each outcome, a multivariable model was constructed for each outcome. We offered to this model variables, which were found with *p* < 0.2 in the preceding univariable analyses. Then, progressively, we removed variables from the model by using backward elimination. The likelihood ratio test was performed to assess *p*-value of each variable; among those found with *p* > 0.2, the one with the largest *p* was removed from the model. We repeated this procedure until we could not remove any value from the model, with *p* > 0.2. The variables included in the final multivariable models constructed for each outcome are detailed in Table S2.

In all analyses, statistical significance was defined at *p* < 0.05.

## 3. Results

### 3.1. Management Patterns during the Mating Season

#### 3.1.1. Mating Patterns

The median month of the start of the mating period was significantly earlier for adult sheep than goats: May (range: February–December) versus June (January–December) (*p* < 0.0001). However, no such difference was evident for replacement animals: in both ewe lambs and replacement goats (‘doelings’), the median month of the start of the mating period was August (range: January–December) (*p* = 0.07) (*p* < 0.0001 compared to adult animals of the respective species).

The median duration of the mating period was similar in adult sheep and goats: 2 (1–12) and 2 (1–12) months (*p* = 0.09), but significantly longer in ewe lambs than in replacement goats: 1 (1–9) versus 1 (1–6) month, respectively (*p* = 0.006). The duration was significantly shorter in the latter animals compared to the adults (*p* < 0.0001).

The management system applied in the farms was not associated with the month of the start (*p* > 0.08) or the duration (*p* > 0.33) of the mating period. With regard to the location of the farms, there was a clear association of farms located more southerly with earlier starting of the mating period, whilst an association of the location with the duration was recorded only in adult ewes. Detailed results are in Tables S3 and S4.

#### 3.1.2. Application of Reproductive Control

Reproductive control was applied in 108 sheep flocks (33.2%, 95% confidence interval (CI): 28.3–38.5%) and 20 goat herds (16.8%, 95% CI: 11.2–24.5%) (*p* = 0.0007 between farms with the two animal species). Mostly, intravaginal progestagen sponges were employed for the practice: in 89 flocks (82.4%) and 16 herds (80.0%); use of melatonin implants was less frequent: in 23 flocks (21.3%) and 5 herds (25.0%) (*p* < 0.0001 for comparison between the two techniques); no use of other techniques was reported.

**Figure 2:**
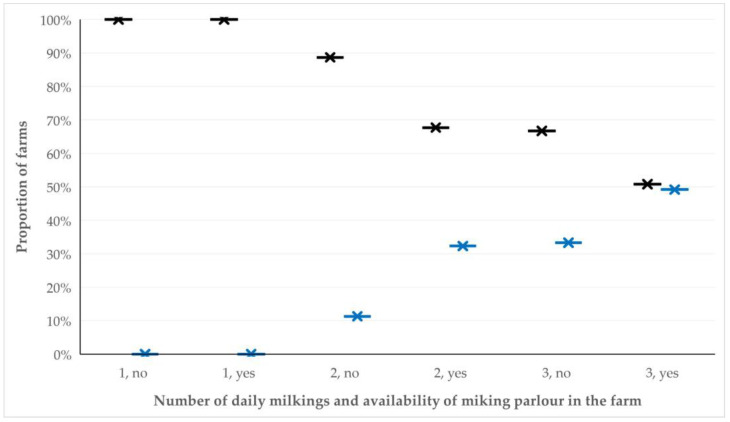
Proportions of small ruminant farms (sheep flocks and goat herds cumulatively) in Greece, in which reproductive control was (blue) or was not (gray) applied, in accord with the number of daily milkings and the availability of milking parlour in the farms.

The results of the univariable analysis for predictors for the application of reproductive control are in Tables S5 and S6. During the multivariable analysis, five predictors emerged as significant in sheep flocks, specifically: (a) availability of milking parlour (*p* = 0.006), (b) number of ewes in a farm (*p* = 0.006), (c) number of daily milkings (*p* = 0.028), (d) daily period spent by farmers at the farm (*p* = 0.035) and (e) farming tradition in the family (*p* = 0.048). In goat herds, two predictors emerged as significant, specifically: (a) number of daily milkings (*p* = 0.009) and (b) availability of milking parlour (*p* = 0.05). Details are in Table 1 and Figure 2.

#### 3.1.3. Use of Male Animals

The median ratio of female to male animals in sheep flocks and goat herds was 22:1 (interquartile range: 12:1) and 25:1 (interquartile range: 13:1), respectively (*p* = 0.63). There was no association between this ratio and the application of reproductive control (*p* > 0.26 for all comparisons).

None of the sheep or goat farmers, 0.0% (0.0–1.2%) and 0.0% (0.0–1.9%), respectively, declared that they used ‘teaser animals’ (animals fitted with teaser harness or animals in which vasectomies had been performed).

#### 3.1.4. Other Procedures

None of the sheep or goat farmers in the study, 0.0% (0.0–1.2%) and 0.0% (0.0–1.9%) respectively, declared that they applied artificial insemination or embryo transfer in their farms.

### 3.2. Management Patterns during Pregnancy

#### 3.2.1. Management of Pregnant Animals

Pregnancy diagnosis by means of ultrasonographic examination was performed in 119 sheep (36.6%, 95% CI: 31.6–42.0%) and 20 goat (16.8%, 95% CI: 11.2–24.5%) farms (*p* < 0.0001). The practice was applied significantly more frequently in farms where reproductive control was also applied: 60.2% and 55.0% versus 24.9% and 9.1% in sheep flocks and goat herds, respectively (*p* < 0.0001 for all comparisons).

The results of the univariable analysis for predictors for pregnancy diagnosis by means of ultrasonographic examination are in Tables S7 and S8. During the multivariable analysis, three predictors emerged as significant in sheep flocks, specifically: (a) management system applied in farms (*p* < 0.0001), (b) age of farmer (*p* = 0.005) and (c) farming tradition in the family (*p* = 0.041). In goat herds, two predictors emerged as significant, specifically: (a) management system applied in farms (*p* = 0.011) and (b) availability of milking parlour (*p* = 0.044). Details are in Table 2.

Most farmers, in sheep and goat farms, modified the nutritional regime during pregnancy, specifically, in 229 sheep (70.5%, 95% CI: 65.3–75.2%) and 68 goat (57.1%, 95% CI: 47.8–66.1%) farms (*p* = 0.008). Additionally, most farmers grouped animals at the end of gestation according to the projected dates of parturition, specifically, in 214 sheep (65.9%, 95% CI: 60.5–70.8%) and 69 goat (58.0%, 95% CI: 49.0–66.5%) farms (*p* = 0.13). Clear associations of the modification of the nutritional regime and the grouping of animals were found with the application of reproductive control and the pregnancy diagnosis by ultrasonographic examination in sheep and goat farms; details are in Tables S9 and S10.

#### 3.2.2. Vaccinations during Pregnancy

**Figure 3:**
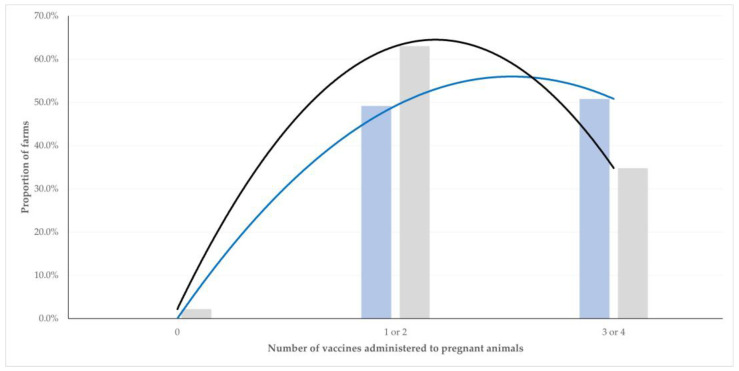
Proportions of small ruminant farms (sheep flocks and goat herds cumulatively) in Greece, in which reproductive control was (blue) or was not (gray) applied, in accord with the number of vaccines administered to animals during pregnancy (*p* = 0.006) (solid lines indicate respective trendlines).

Application of reproductive control was associated with a significantly higher number of vaccines performed during pregnancy in animals in the respective farms compared to animals in the farms where the procedure was not applied: cumulatively, 2.6 ± 0.1 versus 2.2 ± 0.1 vaccines, respectively (*p* = 0.001) (Figure 3). The trend was also seen when sheep and goat farms were considered separately: respectively, 2.6 ± 0.1 versus 2.3 ± 0.1 vaccines and 2.5 ± 0.2 versus 2.1 ± 0.1 vaccines (*p* = 0.014 and *p* = 0.06, respectively).

Similar findings were seen with regard to pregnancy diagnosis by means of ultrasonographic examination. The procedure was associated with a significantly higher number of vaccines performed during pregnancy in animals in the respective farms compared to animals in the farms where the procedure was not applied: cumulatively, 2.7 ± 0.1 versus 2.1 ± 0.1 vaccines, respectively (*p* < 0.0001). The trend was also seen when sheep and goat farms were considered separately: respectively, 2.7 ± 0.1 versus 2.2 ± 0.1 vaccines and 2.6 ± 0.3 versus 2.1 ± 0.1 vaccines (*p* < 0.0001 and *p* = 0.037, respectively).

### 3.3. Management Patterns during the Lambing/Kidding Period

#### 3.3.1. Lambing/Kidding Area

A separate lambing/kidding area was available in 173 sheep (53.2%, 95% CI: 47.8–58.6%) and 58 goat (48.7%, 95% CI: 39.9–57.6%) farms (*p* = 0.40). In approximately half of these farms, this separate area for parturition was a permanent installation: in 49.7% of sheep and 51.7% of goat farms (*p* = 0.79). In sheep farms, a separate area was present more frequently in farms in the northern part of the country (in 61.4% of farms) compared to farms in the central (in 48.2%) or the southern (in 37.1%) part (*p* = 0.010). Moreover, a separate area for parturition was present more frequently in sheep farms managed intensively or semi-intensively (in 58.2% of farms) than in farms managed semi-extensively or extensively (in 46.8%) (*p* = 0.042). In contrast, no such associations were found in goat farms (*p* = 0.60 and *p* = 0.33, respectively).

#### 3.3.2. Induction of Parturition

Application of the induction of parturition was reported by five sheep (1.5%, 95% CI: 0.7–3.6%) and one goat (0.8%, 95% 0.2–4.6%) farmers (*p* = 0.57). In all cases, lambing induction was performed in farms in which reproductive control was also practiced (*p* < 0.0001).

#### 3.3.3. Newborns

The average per farm number of lambs/kids born per ewe/female goat among all farms in the study was 1.33 ± 0.01 for sheep flocks and 1.30 ± 0.02 for goat herds (*p* = 0.15).

The results of the univariable analysis for predictors for the number of lambs/kids born per ewe/female goat are in Tables S11 and S12. During the multivariable analysis, five predictors emerged as significant in sheep flocks, specifically: (a) application of reproductive control (*p* = 0.006), (b) location of the farm (*p* = 0.014), (c) presence of working staff (*p* = 0.030), (d) collaboration with veterinary practice (*p* = 0.031) and (e) breed of ewes (*p* = 0.042). In goat herds, two predictors emerged as significant, specifically: (a) application of reproductive control (*p* < 0.0001) and (b) breed of female goats (*p* = 0.041). Details are in Table 3. With regard to the associations with the specific breeds of sheep and goats, detailed results are in Table S13.

#### 3.3.4. Management Practices at Lambing/Kidding Period

The frequency of the application of other practices in newborn lambs and kids: provision of care to newborns, availability of colostrum bank, newborn fostering, disinfection of umbilicus, tail docking and provision of milk replacer, is shown in Table 4.

Lambs were taken away from their dams (at the age of 50 ± 1 days) earlier than kids from theirs (at the age of 65 ± 3 days) (*p* < 0.0001). Newborns in farms with intensive or semi-intensive management were also taken away from their dams earlier (Table S14).

### 3.4. Replacement of Adult Animals

The average age at which adult sheep were removed from the farm (‘culling age’) was 5.9 ± 0.1 years for ewes and 4.4 ± 0.1 years for rams (*p* < 0.0001). The respective ages for adult goats were 6.9 ± 0.2 years for female goats and 4.9.± 0.2 years for bucks (*p* < 0.0001). The differences between sheep and goats were also significant (*p* < 0.0001 for female animals and *p* = 0.009 for male animals). In both sheep and goat farms, the culling age was earlier in farms with intensive or semi-intensive management (*p* < 0.010 for all comparisons) (Table S15).

In most cases, farmers sourced replacement animals exclusively from their own farms (54.2%), with fewer farmers exclusively purchasing the replacement animals (6.6%), whilst 39.2% of farmers obtained animals from both sources. In both sheep and goat farms, milk production of the dam was the criterion employed for maintaining replacement animals (Table 5). Again, in both sheep and goat farms, milk production was the criterion employed for selecting replacement animals for purchase (Table 6).

### 3.5. Associations with Production Parameters

No associations were found between the start of the mating period and the various production parameters assessed (*p* ≥ 0.06) (Table S16).

With regard to the application of reproductive control, there was a clear association for increased milk production and the number of newborns per female animal in sheep and goat farms that applied the procedure (*p* ≤ 0.07). For the other parameters, no associations were seen (*p* > 0.15) (Table S17).

With regard to pregnancy diagnosis by means of ultrasonographic examination, there was a clear significance for increased milk production and the number of newborns per female animal in sheep and goat farms that applied the procedure (*p* < 0.03). For the other parameters, no associations were seen (*p* > 0.13), bar for protein content in sheep flocks for which a significance was found (*p* = 0.004) (Table S18).

With regard to the age when newborns were taken away from their dams, an inverse correlation was seen with milk production and a positive correlation with fat content in the milk (*p* ≤ 0.045). There was also some correlation between the age when newborns were taken away from their dams and the somatic cell counts in the bulk tank milk (*p* = 0.028 in sheep flocks, *p* = 0.09 in goat herds). For the other parameters, no associations were seen (*p* ≥ 0.13) (Table S19).

**Figure 4:**
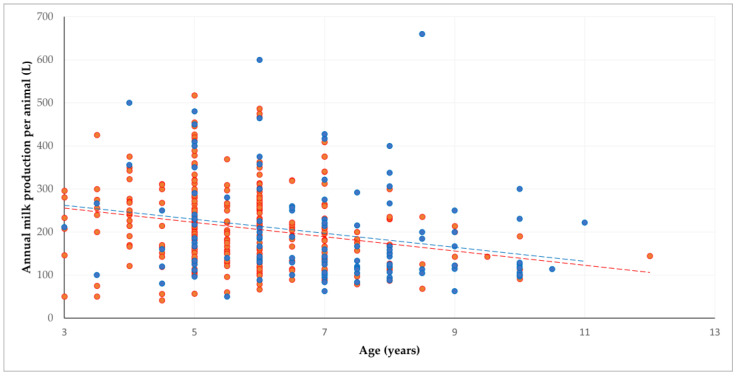
Inverse correlation between age in which adult animals were replaced, and milk production (red dots: sheep farms, blue dots: goat farms; dashed lines indicate respective trendlines).

With regard to the age in which adult animals were replaced, an inverse correlation was seen with milk production (*p* < 0.01) (Figure 4). There was also an inverse correlation with the average number of kids born per female goat (*p* = 0.034). For the other parameters, no associations were seen (*p* ≥ 0.09) (Table S20).

In goat farms, in which replacement animals were sourced from those same farms, higher milk production was seen in the farms where the milkability was the selection criterion (*p* = 0.030). For the other comparisons, no significances were seen (*p* ≥ 0.32) (Table S21).

## 4. Discussion

### 4.1. Preamble

In this work, we studied the patterns of reproductive management applied in 444 small ruminant farms (325 sheep flocks and 119 goat herds) during an extensive countrywide investigation in Greece. During this work, we also assessed potential associations of the patterns of reproductive management with production outcomes; moreover, we included in our evaluation the human resources available on the farms. The study involved a large number of farms, in all regions of the country; hence, conditions prevailing throughout the country were taken into account and factors of regional importance weighed less. As far as we are aware, this is the largest sample size ever employed internationally. Whilst the importance of reproductive management in high production in sheep and goat farms is widely recognised, there is a paucity of detailed relevant information. Hence, the results provide a useful guide regarding reproductive management performed in sheep and goat farms in Greece.

According to data sourced from the Hellenic Milk Board [5], the farms included in this study refer to approximately 1% of the total number of sheep and goat farms in Greece. Farms were included in the study on a convenience basis, but the approach employed guaranteed that farmers would accept the visit, whilst the presence of an accompanying local veterinarian contributed to minimising suspiciousness and distrust from their part, which consequently led to a relaxed interview. This approach supported the inclusion of flocks and herds with farmers genuinely willing to participate in the study and provide thoughtful and correct answers. There was some stratification in the selection of the farms in the study, as the flocks and herds visited were located in all 13 administrations of the country.

During the study, we used consistent methodologies and ensured that the interviews were always performed by the same investigator (author D.T.L.). Whilst the general limitations of questionnaire surveys applied in this work (e.g., unconscientious responses by farmers and differences in understanding and interpretation of the question) [12], we tried our utmost to decrease any possible adverse effects in the study; for example, queries of the respondents were answered immediately by the interviewer (author D.T.L.), and, at the same time, the principal author (author G.C.F.) discussed some of the answers of the interviewees with the veterinarians accompanying them at the farms, with the objective to verify the accuracy of the responses provided.

### 4.2. Mating Period and Application of Reproductive Control

The findings are in accord with those expected: the start of the mating season in May in sheep flocks, i.e., at the end of the spring, would be typical for locations in the geographical latitude of Greece [13], whilst for goats, the mating season started one month later. The later start in replacement animals is also consistent with the delayed attainment of puberty in those animals, which coincides with the end of the summer or early autumn.

The start of the mating season in May/June (sheep/goats, respectively) may be responsible for the limited application of reproductive control, as there is no significant need for an earlier start of the breeding period. The more extensive use of progestogen sponges, compared to the use of melatonin implants, may possibly be due to the complicated protocol required for the use of the latter technique.

We postulate that the identification of the availability of a milking parlour and the number of daily milkings as common predictors, in sheep and goat farms, for using reproductive control, suggests that these two factors could be related to an easier application of the technique. In this context, the milking parlour would be used to walk through the animals, whilst the frequent milking sessions would provide more opportunities to gather the animals in order to apply the techniques.

The findings indicate a clear benefit for the application of reproductive control, in terms of a higher number of lambs and kids born, as well as increased milk production. In Greece, no specific financial assessment of the scheme has been made, although Gelasakis [14] considered the application of reproductive control as important for profit making in sheep and goat farming. A more precise assessment was made in Jordan by Nasr et al. [15], who reported a profit of USD 8.36 per ewe in flocks in which reproductive control had been applied. Specific financial benefits in reproductive control were also reported by Yu et al. [16]. It should be noted that in Greece, prices for sheep and goat milk would be higher (by about 0.03–0.05 Euros) in late summer and early autumn, when animals subjected to reproductive control would be lambing/kidding, due to the limited quantities of milk available at the time. Moreover, animals can continue to graze until October to early November due to the generally mild weather and availability of grass, thus contributing to reduced feeding costs in these flocks/herds during pregnancy and early lactation.

### 4.3. Pregnancy Diagnosis by Ultrasonographic Examination

Ultrasonographic examination of the pregnant uterus is a simple, reliable, non-invasive and non-disruptive imaging technique. It is safe for the animal under examination and the operator. Its objective is the achievement of an accurate diagnosis of pregnancy early during the gestation period [17,18]. Further ultrasonographic examinations can be performed to identify multiple pregnancies and count the number of foetuses borne, to identify the sex of the foetuses and estimate their viability and to assess foetal growth [19]. Pregnancy diagnosis by ultrasonographic examination contributes to improved health management of ewes and female goats during pregnancy [20].

The identification of the intensive or semi-intensive management system in a farm as a predictor for the application of the technique is in line with the frequent use of the technique in these farms to facilitate the correct application of various practices often followed under such management, e.g., grouping of ewes according to the stage of gestation, high-energy feeding at the final stage of that period, hiring of part-time staff for the lambing/kidding season. The cost of the examination can be compensated by the higher milk production and higher number of lambs/kids born, as found in farms where the practice was applied.

A significant benefit of applying reproductive control and carrying out pregnancy diagnosis by ultrasonographic examination was the association found with the increased number of vaccines administered in the respective farms, which would afford increased protection against the respective infections in dams and newborns. In sheep and goats, the gestation period is used for the administration of vaccines against clostridial infections and bacterial pneumonia, in order to provide protection to the female animals, as well as to their offspring. In dairy production systems, there is also a need to vaccinate against two significant mammary infections, staphylococcal mastitis and contagious agalactia [8], and therefore the number of vaccines for potential administration to pregnant ewes and female goats would be higher. In farms where these two management practices are applied, the expected dates for lambing/kidding season can be found out with higher accuracy and, consequently, the administration of vaccines to the animals can be planned better, thus avoiding the cost for carrying out repeat vaccinations in cases of unknown lambing/kidding dates [20].

### 4.4. Number of Newborn Lambs/Kids

For imported sheep and goat breeds, as well as for the Chios sheep breed (animals of which are considered to produce a high number of lambs), the number of newborns reported by the farmers was smaller than described in the literature [21,22]. In contrast, for the other Greek breeds, which are considered to produce small numbers of newborns, the present results were similar to those in the literature [21,22]. A reason for this discrepancy can be that relevant studies had been performed in research establishments, where animals were under ideal conditions (housing, health management, feeding etc.) and thus exhibited their full potential. In contrast, under commercial conditions, the number of newborns was, in reality, smaller.

The genetic background of animals has been well documented as an important determinant of fecundity and prolificacy in sheep and goats [23,24,25]; thus, it was reasonable for the animal breed to emerge as a predictor for the number of newborns in the present study. Moreover, reproductive control protocols include the administration of hormones (e.g., equine chorionic gonadotrophin, which increases the number of follicles that would ultimately ovulate [26,27]), and thus was found to be associated with a higher number of newborns as well.

The reason for the inverse correlation of the age when newborns are taken away from their dam with increased milk production is obvious: a shorter suckling period leads to a longer milking period; hence, higher milk production. The positive association of the age when newborns are taken away from their dam with higher somatic cell counts can be explained as the result of the higher incidence of mastitis, which may occur during sucking by the lambs or kids, given that sucking contributes to the transfer of bacteria from the mouths of lambs to the teats of ewes [28,29] and also leads to teat lesions, for which there is field evidence suggesting that they predispose ewes to mastitis [30]. Ultimately, these would be reflected in higher somatic cell counts in milk.

The availability of a separate lambing/kidding area in farms is recommended, in order to increase the survival of newborn lambs/kids and for establishing a dam–offspring bond [31]. Nevertheless, recent findings have indicated an increased risk to farmers for infection with *Brucella melitensis* [32]; hence, the need to implement increased biosecurity measures in such cases must be stressed.

### 4.5. Culling and Replacement of Ewes/Female Goats

Culling is implemented to increase the efficiency of sheep and goat farms. The usual criteria for culling animals include suboptimal reproductive performance, poor milk production and chronic-type health problems.

In general, the culling age for ewes is 5 to 6 years (which results in a replacement rate of 15–20% annually) (Australia: Hassall and Associates [33]; New Zealand: Farrell et al. [34]), whilst the age of culling of rams has been found to be 3.5 to 3.8 years (Spain: Mozo et al. [35]; United Kingdom: Phillips et al. [36]). The findings of the present study are aligned with those in the literature.

However, the relevant results for goats indicate a difference to our findings: the study of Malher et al. [37] (France) indicated a culling age of 6 years for female goats. The reason for this may be that in France, the management of goat farms is under more intensive management. In farms in the current study, the culling age of goats was found to be lower (6.3 years in herds with intensive or semi-intensive management versus 7.2 years in herds with semi-extensive or extensive management).

The inverse association of culling age with milk production reflects the progressive decrease in milk production of ewes and female goats with age [13,34] and coincides with the increasing culling rate in those animals.

The association of milkability (i.e., the ability of an animal to give a regular, complete and rapid milk secretion by the mammary gland in response to milking [38]) with higher milk production may possibly be the result of emptying completely the mammary glands during milking in animals with a well-formed and easily-milked udder. This was significant in goats, a species in which machine milking presents some challenges, as milking systems are manufactured primarily for sheep [39].

## 5. Conclusions

The study explored the patterns of reproductive management in small ruminant farms (sheep flocks and goat herds). The correct implementation of reproductive management in small ruminant farms, based on scientific principles and compliance with established regulations, is important for the improvement in the welfare of farm animals and high production outcomes as shown in this study.

The findings provide a detailed report of the reproductive management practices followed in sheep and goats in Greece. The results also indicate an association of reproductive practices with production outcomes, specifically, milk production and the number of lambs/kids born. There is a possibility for increasing the use of some practices, e.g., the control of reproduction and pregnancy diagnosis by ultrasonographic examination, which would contribute to improving the health management in the farms. This was shown by the increased number of vaccines administered in farms where these practices were applied, as well as the higher production outcomes in these farms.

## Figures and Tables

**Table 1:** Multivariable analysis for predictors for application of reproductive control in sheep and goat farms in Greece.

Variables	Odds Ratio ^1^ (95% Confidence Intervals)	*p*-Value
**Sheep Flocks**		
Availability of milking parlour		0.006
Yes (97/255 = 38.0% ^2^)	3.293 (1.649–6.575)	0.0007
No (11/70 = 15.7%)	reference	-
Number of ewes in a farm		0.006
Per unit decrease	8 (0–17)	0.006
Number of daily milkings		0.028
One (0/1 = 0.0%)	reference	-
Two (80/264 = 30.3%)	1.309 (0.053–32.478)	0.87
Three (28/60 = 46.7%)	2.631 (0.103–67.170)	0.56
Daily period spent by farmers at the farm		0.035
≤8 h (44/99 = 44.4%)	2.023 (1.240–3.308)	0.005
>8 h (64/226= 28.3%)	reference	-
Farming tradition in the family		0.048
Yes (87/283 = 30.7%)	reference	-
No (21/42= 50.0%)	2.253 (1.170–4.339)	0.015
**Goat Herds**		
Number of daily milkings		0.009
One (0/4 = 0.0%)	reference	-
Two (16/108 = 14.8%)	1.605 (0.083–31.242)	0.75
Three (4/7 = 57.1%)	11.571 (0.454–295.032)	0.14
Availability of milking parlour		0.05
Yes (16/66 = 24.2%)	3.920 (1.224–12.559)	0.022
No (4/53 = 7.5%)	reference	-

^1^ Odds ratios calculated against the lowest prevalence associations of the variable. ^2^ Numerator: no. of farms among those included in the denominator, in which the outcome of interest was seen; denominator: no. of farms in which the studied variable prevailed.

**Table 2:** Multivariable analysis for predictors for pregnancy diagnosis by means of ultrasonographic examination in sheep and goat farms in Greece.

Variables	Odds Ratio ^1^ (95% Confidence Intervals)	*p*-Value
**Sheep Flocks**		
Management system applied in farms		<0.0001
Intensive or semi-intensive (90/184 = 48.9% ^2^)	3.700 (2.242–6.099)	<0.0001
Semi-extensive or extensive (29/141 = 20.6%)	reference	-
Age of farmer		0.005
≤50 years (91/197 = 46.2%)	3.066 (1.852–5.076)	<0.0001
>50 years (28/128 = 21.9%)	reference	-
Farming tradition in the family		0.041
Yes (90/283 = 31.8%)	reference	-
No (29/42 = 69.0%)	4.784 (2.375–9.637)	<0.0001
**Goat Herds**		
Management system applied in farms		0.011
Intensive or semi-intensive (12/38 = 31.6%)	4.212 (1.549–11.452)	0.005
Semi-extensive or extensive (8/81 = 9.9%)	reference	-
Availability of milking parlour		0.044
Yes (18/66 = 27.3%)	9.563 (2.106–43.423)	0.003
No (2/53 = 3.8%)	reference	-

^1^ Odds ratios calculated against the lowest prevalence associations of the variable. ^2^ Numerator: no. of farms among those included in the denominator, in which the outcome of interest was seen; denominator: no. of farms in which the studied variable prevailed.

**Table 3:** Multivariable analysis for predictors for high number of lambs/kids born per ewe/female goat in sheep and goat farms in Greece.

Variables	Odds Ratio ^1^ (95% Confidence Intervals)	*p*-Value
**Sheep Flocks**		
Application of reproductive control		0.006
Yes (51/108 = 47.2% ^2^)	1.390 (0.872–2.214)	0.17
No (85/217 = 39.2%)	reference	-
Location of farm		0.014
Northern part of Greece (82/153 = 53.6%)	2.525 (1.561–4.083)	0.0002
Central part of Greece (43/137 = 31.4%)	reference	-
Southern part of Greece (11/35 = 31.4%)	1.002 (0.450–2.229)	0.99
Presence of working staff		0.030
Yes (59/123 = 48.0%)	1.497 (0.951–2.356)	0.08
No (77/202 = 38.1%)	reference	-
Collaboration with veterinary practice		0.031
Yes (123/283 = 43.5%)	1.656 (0.823–3.329)	0.16
No (13/41 = 31.7%)	reference	-
Breed of ewes		0.042
Crossbreeds (12/43 = 27.9%)	reference	-
Imported breeds (70/139 = 50.4%)	2.621 (1.245–5.519)	0.011
Local breeds (54/143 = 37.8%)	1.567 (0.742–3.309)	0.24
**Goat Herds**		
Application of reproductive control		<0.0001
Yes (14/20 = 70.0%)	5.118 (1.797–14.575)	0.002
No (31/99 = 31.3%)	reference	-
Breed of ewes		0.041
Crossbreeds (9/18 = 50.0%)	3.000 (0.994–9.052)	0.05
Imported breeds (22/45 = 48.9%)	2.870 (1.237–6.655)	0.014
Local breeds (14/56 = 25.0%)	reference	-

^1^ Odds ratios calculated against the lowest prevalence associations of the variable. ^2^ Numerator: no. of farms among those included in the denominator, in which the outcome of interest was seen; denominator: no. of farms in which the studied variable prevailed.

**Table 4:** Frequency of carrying out various practices related to management of newborns in sheep and goat farms in Greece.

Practice	Sheep Flocks	Goat Herds	*p*
Provision of care to newborns	293 (90.2%, 95% CI: 86.4–92.9%	109 (91.6%, 95% CI: 85.2–95.4%)	0.65
Availability of colostrum bank	46 (14.2%, 95% CI: 10.8–18.4%)	12 (10.1%, 95% CI: 5.9–16.8%)	0.26
Practicing new- born fostering	112 (34.5%, 95% CI: 29.5–39.8%)	86 (72.3%, 95% CI: 63.6–79.5%	<0.0001
Disinfection of navel stump	213 (65.5%, 95% CI: 60.2–70.5%)	65 (54.6%, 95% CI: 45.7–63.3%)	0.035
Tail docking	244 (75.1%, 95% CI: 70.1–79.6%)	34 (28.6%, 95% 21.2–37.3%)	<0.0001
Provision of milk replacer	22 6.8% (95% CI: 4.5–10.0%)	1 0.8% (95% CI: 0.2–4.6%)	0.013

**Table 5:** Criteria evaluated by farmers for sourcing replacement animals from their own farms.

Criteria	Sheep Flocks	Goat Herds	*p*
Dam milk production	254 (84.7%)	98 (88.3%)	0.35
Dam prolificacy	2 (0.7%)	1 (0.9%)	0.84
General animal morphology	54 (18.0%)	27 (24.3%)	0.15
Introduction of new pedigree	8 (2.7%)	1 (0.9%)	0.27
Milkability	65 (21.7%)	21 (18.9%)	0.54

**Table 6:** Criteria evaluated by farmers for selecting replacement animals for purchase for their farms.

Criteria	Sheep Flocks	Goat Herds	*p*
Milk production records	95 (62.5%)	20 (55.6%)	0.44
General animal morphology	17 (11.2%)	7 (19.4%)	0.18
Introduction of new pedigree	46 (30.3%)	11 (30.6%)	0.97
Potential milkability	34 (22.4%)	13 (36.1%)	0.09
Animal breed	1 (0.7%)	0 (0.0%)	0.62

## Data Availability

Most data presented in this study are in the Supplementary Materials. The remaining data are available on request from the corresponding author. The data are not publicly available as they form part of the PhD thesis of the first author, which has not yet been examined, approved and uploaded in the official depository of PhD theses from Greek Universities.

## Supplementary Materials

The following supporting information can be downloaded at: https://www.mdpi.com/article/10.3390/ani12243455/s1, Table S1: Details of variables (n = 48) collected during interviews of farmers by means of a structured questionnaire and used in the evaluations for potential associations with patterns of reproductive management in 325 sheep flocks and 199 goat herds during a countrywide investigation in Greece; Table S2: Details of multivariable models (n = 6) employed for the evaluation for reproductive management procedures in 325 sheep flocks and 119 goat herds in Greece; Table S3: Month (median (range)) of start of mating season in sheep and goat farms in Greece, in accord with the management system in the farms or the location of the farms in the country; Table S4: Duration (months) (median (range)) of start of mating season in sheep and goat farms in Greece, in accord with the management system in the farms or the location of the farms in the country; Table S5: Results of univariable analysis for associations with application of reproductive control in 325 sheep flocks in Greece; Table S6: Results of univariable analysis for associations with application of reproductive control in 119 goat herds in Greece; Table S7: Results of univariable analysis for associations with pregnancy diagnosis by means of ultrasonographic examination in 325 sheep flocks in Greece; Table S8: Results of univariable analysis for associations with pregnancy diagnosis by means of ultrasonographic examination in 119 goat herds in Greece; Table S9: Associations of the modification of the nutritional regime during pregnancy and the grouping of animals at the end of gestation according to the projected dates of parturition with application of reproductive control in 325 sheep flocks and 119 goat herds in Greece; Table S10: Associations of the modification of the nutritional regime during pregnancy and the grouping of animals at the end of gestation according to the projected dates of parturition with pregnancy diagnosis by means of ultrasonographic examination in 325 sheep flocks and 119 goat herds in Greece; Table S11: Results of univariable analysis for associations with number of lambs born per ewe in 325 sheep flocks in Greece; Table S12: Results of univariable analysis for associations with number of kids born per female goat in 119 goat herds in Greece; Table S13: Number of lambs/kids born per ewe/female goat in accord with breeds of animals in sheep and goat farms in Greece; Table S14: Age of lambs/kids taken away from dam in accord with management system in sheep and goat farms in Greece: Table S15: Age of replacement of adult animals in accord with management system in sheep and goat farms in Greece; Table S16: Associations between the start of the mating period and production parameters assessed in sheep and goat farms in Greece; Table S17: Associations between the application of reproductive control and production parameters assessed in sheep and goat farms in Greece; Table S18: Associations between pregnancy diagnosis by means of ultrasonographic examination and production parameters assessed in sheep and goat farms in Greece; Table S19: Correlations between the age that newborns were taken away from their dams and production parameters assessed in sheep and goat farms in Greece; Table S20: Correlations between the age that adult animals were removed from the farm and production parameters assessed in sheep and goat farms in Greece; Table S21: Associations between criteria evaluated by farmers for sourcing replacement animals from their own farms and production parametres assessed in sheep and goat farms in Greece.
