# Supplementary material for: Patterns of Reproductive Management in Sheep and Goat Farms in Greece
Source: Animals (Basel). 2022 Dec 7;12(24):3455. doi: 10.3390/ani12243455 (PMC9774088; doi:10.3390/ani12243455)
Supplement: Supplementary file 1 [file animals-12-03455-s001.zip › animals-2057923-SI.pdf]

Patters of Reproductive Management in Sheep and Goat Farms in Greece

Daphne T. Lianou, Natalia G.C. Vasileiou, Charalambia K. Michael, Irene Valasi, Vasia S. Mavrogianni, Mariangela Caroprese and George C. Fthenakis

Table S1. Details of variables ($n = 48$) collected during interview of farmers by means of a structured questionnaire and used in the evaluations for potential associations with patterns of reproductive management in 325 sheep flocks and 199 goat herds during a countrywide investigation in Greece.

Management system applied in the farm (description according to EFSA classification [1])
Total grazing land by the farm animals (acres)
Type of milking (hand-milking / machine-milking)
Daily number of milking sessions (no.)
No. of female animals in the farm (no.)
No. of male animals in the farm (no.)
Breed of animals in the farm (description)
Average age of culling female animals (years)
Source of replacement animals (own animals / purchase)
Criteria for selection of own animals as replacements (description)
Criteria for selection of animals for purchase as replacements (description)
Total milk quantity per ewe / doe obtained during the preceding milking period (litres)
Total number of lambs / kids born during the preceding lambing season (no.)
Collaboration with a veterinarian (yes / no)
Beginning of the mating period for ewes / female goats (month)
End of the mating period for ewes / female goats (month)
Beginning of the mating period for ewe-lambs and replacement female goats (month)
End of the mating period for ewe-lambs and doelings (month)
Reproductive management (no hormonal control / administration of melatonin / administration of progestogens / application of other techniques)
Use of teaser male animals (yes / no)
Use of artificial insemination (yes / no)
Use of embryo transfer (yes / no)
Use of ultrasound for pregnancy diagnosis (yes / no)
Nutritional modifications before the lambing period (yes / no)
Grouping of pregnant females during the final stage of pregnancy (yes / no)
Induction of lambing (yes / no)
Availability of a separate lambing / kidding area (yes / no)
Newborn care and specific monitoring (yes / no)
Maintenance of a colostrum bank (yes / no)
Newborn fostering to female animals other than their dams (yes / no)

Disinfection of the navel stump in newborns (yes / no)
Tail docking in newborns (yes / no)
Administration of milk replacer to lambs (yes / no)
Vaccination against clostridial infections (yes / no)
Vaccination regime applied (description)
Vaccination against contagious agalactia (yes / no)
Vaccination regime applied (description)
Vaccination against pneumonia (yes / no)
Vaccination regime applied (description)
Vaccination against staphylococcal mastitis (yes / no)
Vaccination regime applied (description)
Age of farmer (years)
Length of previous animal farming experience (years)
Farmer's general education (description: primary = European Qualifications Framework Levels 1 or 2, secondary = or post-secondary = European Qualifications Framework Levels 3, 4 or 5, tertiary = European Qualifications Framework Level 6, 7 or 8)
Farmer's professional involvement in farming (full-time / part-time)
Daily period spent by farmer at the farm (hours)
Family tradition in farming (yes / no)
Presence of working staff in the farm (yes/no)

[1] management system classified as intensive, semi-intensive, semi-extensive, extensive (European Food Safety Authority. Scientific opinion on the welfare risks related to the farming of sheep for wool, meat and milk production. *EFSA J.* **2014**, *12*, 3933–4060.).

Table S2. Details of multivariable models (n = 6) employed for the evaluation for reproductive management procedures in 325 sheep flocks and 119 goat herds in Greece.

Outcome	Variables (n)		
	**assessed in univariable analyses**	**offered to the multivariable models**	**required in the final models**
Application of reproductive control - sheep	16	10	(a) Availability of milking parlour, (b) No. of ewes in the flock, (c) Number of daily milking sessions, (d) Age of farmer, (e) Daily period spent by farmer at the farm, (f) Family tradition in farming
Application of reproductive control - goats	16	5	(a) Availability of milking parlour, (b) Number of daily milking sessions, (c) Daily period spent by farmer at the farm
Pregnancy diagnosis by means of ultrasonographic examination - sheep	11	9	(a) Management system applied in farm, (b) Availability of milking parlour, (c) Age of farmer, (d) Length of previous animal farming experience of the farmer, (e) Family tradition in farming
Pregnancy diagnosis by means of ultrasonographic examination - goats	11	8	(a) Management system applied in farm, (b) Availability of milking parlour
High number of lambs born per ewe	16	6	(a) Location of the farm, (b) Breed of animals, (c) Collaboration with a veterinarian, (d) Application of reproductive control, (e) Presence of working staff in the flock
High number of kids born per female goat	16	7	(a) Breed of animals, (b) Application of reproductive control

Table S3. Month (median (range)) of start of mating season in sheep and goat farms in Greece, in accord with the management system in the farms or the location of the farms in the country.

Management system	Sheep		Goats	
	**Adult ewes**	**Ewe-lambs**	**Adult does**	**Doelings**
Intensive or semi-intensive	May (Feb.-Oct.)	August (Jan.-Dec.)	June (Mar.-Oct.)	August (Jan.-Dec.)
Semi-extensive or extensive	May (Feb.-Dec.)	August (May.-Dec.)	June (Jan.-Dec.)	August (Jan.-Dec.)
p	0.88	0.09	0.68	0.92
**Area of the country, where farms are located**	**Sheep**		**Goats**	
	**Adult ewes**	**Ewe-lambs**	**Adult does**	**Doelings**
Northern part	June (Mar.-Sep.)	August (Jan.-Dec.)	July (Mar.-Sep.)	September (Jan.-Dec.)
Central part	May (Feb.-Oct.)	August (Jan.-Dec.)	May (Feb.-Dec.)	August (Jan.-Dec.)
Southern part	May (Apr.-Dec.)	August (May.-Dec.)	May (Jan.-Jul.)	July (Jan.-Nov.)
p	< 0.0001	0.017	< 0.0001	0.09

Table S4. Duration (months) (median (range)) of start of mating season in sheep and goat farms in Greece, in accord with the management system in the farms or the location of the farms in the country.

**Management system**	**Sheep**		**Goats**	
	**Adult ewes**	**Ewe-lambs**	**Adult does**	**Doelings**
Intensive or semi-intensive	2 (1-12)	1 (0-9)	1.5 (1-12)	1 (0-5)
Semi-extensive or extensive	3 (1-12)	1 (0-7)	2 (1-12)	1 (0-6)
p	0.54	0.44	0.34	0.38
**Area of the country, where farms are located**	**Sheep**		**Goats**	
	**Adult ewes**	**Ewe-lambs**	**Adult does**	**Doelings**
Northern part	2 (1-12)	1 (0-8)	1.5 (1-7)	1 (0-6)
Central part	3 (1-12)	1 (0-9)	2 (1-12)	1 (0-5)
Southern part	1 (1-12)	1 (0-5)	1 (1-12)	1 (0-3)
p	0.030	0.20	0.88	0.18

Table S5. Results of univariable analysis for associations with application of reproductive control in 325 sheep flocks in Greece.

**Reproductive control performed (n = 108)**			**Reproductive control not performed (n = 217)**			***p***
**Location of the farm**						
Northern part of Greece	Central part of Greece	Southern part of Greece	Northern part of Greece	Central part of Greece	Southern part of Greece	
49	47	12	104	90	23	0.91
**Management system applied in the farm**						
Intensive or Semi-intensive	Semi-extensive or Extensive		Intensive or Semi-intensive	Semi-extensive or Extensive		
70	38		114	103		0.035
**Availability of a separate lambing area**						
Yes	No		Yes	No		
59	49		114	103		0.72
**Total land available for grazing**						
1.47 ± 0.27 acres per animal			2.50 ± 0.48 acres per animal			0.14
**Availability of milking parlour**						
Yes	No		Yes	No		
97	11		158	59		0.0004
**No. of ewes in the flock**						
285 ± 24			344 ± 16			0.038
**Breed of animals**						
Crossbreeds	Imported breeds	Local breeds	Crossbreeds	Imported breeds	Local breeds	
16	49	43	27	89	101	0.51
**Collaboration with a veterinarian**						
Yes	No		Yes	No		
99	9		184	33		0.08

**Number of daily milking sessions**						
One	Two	Three	One	Two	Three	
0	80	28	1	184	32	0.041
**Age of the farmer**						
Up to 50 years	Over 50 years		Up to 50 years	Over 50 years		
76	32		121	96		0.011
**Length of previous animal farming experience of the farmer**						
≤ 5 years	> 5 years		≤ 5 years	> 5 years		
34	74		40	177		0.008
**Education of the farmer**						
Primary education	Secondary or post-secondary education	Tertiary education	Primary education	Secondary or post-secondary education	Tertiary education	
21	71	16	36	154	27	0.63
**Professional involvement in farming**						
Full-time	Part-time		Full-time	Part-time		
95	13		197	20		0.43
**Daily period at the farm**						
≤ 8 hours	> 8 hours		≤ 8 hours	> 8 hours		
44	64		55	162		0.005
**Family tradition in farming**						
Yes	No		Yes	No		
87	21		196	21		0.013
**Presence of working staff in the flock**						
Yes	No		Yes	No		
39	69		85	132		0.59

Table S6. Results of univariable analysis for associations with application of reproductive control in 119 goat herds in Greece.

**Reproductive control performed (*n* = 20)**			**Reproductive control not performed (*n* = 99)**			***p***
**Location of the farm**						
Northern part of Greece	Central part of Greece	Southern part of Greece	Northern part of Greece	Central part of Greece	Southern part of Greece	
8	10	2	44	38	17	0.56
**Management system applied in the farm**						
Intensive or Semi-intensive	Semi-extensive or Extensive		Intensive or Semi-intensive	Semi-extensive or Extensive		
9	11		29	70		0.17
**Availability of a separate kidding area**						
Yes	No		Yes	No		
9	11		49	50		0.71
**Total land available for grazing**						
6.43 ± 3.26 acres per animal			6.69 ± 1.10 acres per animal			0.93
**Availability of milking parlour**						
Yes	No		Yes	No		
16	4		50	49		0.015
**No. of does in the herd**						
218 ± 49			249 ± 23			0.68
**Breed of animals**						
Crossbreeds	Imported breeds	Local breeds	Crossbreeds	Imported breeds	Local breeds	
2	12	6	16	33	50	0.08
**Collaboration with a veterinarian**						
Yes	No		Yes	No		
18	2		83	16		0.48

<table>
<tr><td colspan="12">Number of daily milking sessions</td><td></td></tr>
<tr><td colspan="2">One</td><td colspan="2">Two</td><td colspan="2">Three</td><td colspan="2">One</td><td colspan="2">Two</td><td colspan="2">Three</td><td></td></tr>
<tr><td colspan="2">0</td><td colspan="2">16</td><td colspan="2">4</td><td colspan="2">4</td><td colspan="2">92</td><td colspan="2">3</td><td>0.010</td></tr>
<tr><td colspan="12">Age of the farmer</td><td></td></tr>
<tr><td colspan="3">Up to 50 years</td><td colspan="3">Over 50 years</td><td colspan="3">Up to 50 years</td><td colspan="3">Over 50 years</td><td></td></tr>
<tr><td colspan="3">18</td><td colspan="3">2</td><td colspan="3">55</td><td colspan="3">4</td><td>0.64</td></tr>
<tr><td colspan="12">Length of previous animal farming experience of the farmer</td><td></td></tr>
<tr><td colspan="3">≤ 5 years</td><td colspan="3">> 5 years</td><td colspan="3">≤ 5 years</td><td colspan="3">> 5 years</td><td></td></tr>
<tr><td colspan="3">6</td><td colspan="3">14</td><td colspan="3">18</td><td colspan="3">81</td><td>0.23</td></tr>
<tr><td colspan="12">Education of the farmer</td><td></td></tr>
<tr><td colspan="2">Primary education</td><td colspan="2">Secondary or post-secondary education</td><td colspan="2">Tertiary education</td><td colspan="2">Primary education</td><td colspan="2">Secondary or post-secondary education</td><td colspan="2">Tertiary education</td><td></td></tr>
<tr><td colspan="2">1</td><td colspan="2">18</td><td colspan="2">1</td><td colspan="2">19</td><td colspan="2">71</td><td colspan="2">9</td><td>0.22</td></tr>
<tr><td colspan="12">Professional involvement in farming</td><td></td></tr>
<tr><td colspan="3">Full-time</td><td colspan="3">Part-time</td><td colspan="3">Full-time</td><td colspan="3">Part-time</td><td></td></tr>
<tr><td colspan="3">18</td><td colspan="3">2</td><td colspan="3">87</td><td colspan="3">12</td><td>0.79</td></tr>
<tr><td colspan="12">Daily period at the farm</td><td></td></tr>
<tr><td colspan="3">≤ 8 hours</td><td colspan="3">> 8 hours</td><td colspan="3">≤ 8 hours</td><td colspan="3">> 8 hours</td><td></td></tr>
<tr><td colspan="3">7</td><td colspan="3">13</td><td colspan="3">20</td><td colspan="3">79</td><td>0.15</td></tr>
<tr><td colspan="12">Family tradition in farming</td><td></td></tr>
<tr><td colspan="3">Yes</td><td colspan="3">No</td><td colspan="3">Yes</td><td colspan="3">No</td><td></td></tr>
<tr><td colspan="3">16</td><td colspan="3">4</td><td colspan="3">87</td><td colspan="3">12</td><td>0.35</td></tr>
<tr><td colspan="12">Presence of working staff in the flock</td><td></td></tr>
<tr><td colspan="3">Yes</td><td colspan="3">No</td><td colspan="3">Yes</td><td colspan="3">No</td><td></td></tr>
<tr><td colspan="3">5</td><td colspan="3">15</td><td colspan="3">29</td><td colspan="3">70</td><td>0.70</td></tr>
</table>

Table S7. Results of univariable analysis for associations with pregnancy diagnosis by means of ultrasonographic examination in 325 sheep flocks in Greece.

<table>
<tr><th colspan="6">Pregnancy diagnosis performed (n = 119)</th><th colspan="6">Reproductive control not performed (n = 206)</th><th>p</th></tr>
<tr><td colspan="13">Management system applied in the farm</td></tr>
<tr><td colspan="3">Intensive or Semi-intensive</td><td colspan="3">Semi-extensive or Extensive</td><td colspan="3">Intensive or Semi-intensive</td><td colspan="3">Semi-extensive or Extensive</td><td></td></tr>
<tr><td colspan="3">90</td><td colspan="3">29</td><td colspan="3">94</td><td colspan="3">112</td><td><0.0001</td></tr>
<tr><td colspan="13">Availability of milking parlour</td></tr>
<tr><td colspan="3">Yes</td><td colspan="3">No</td><td colspan="3">Yes</td><td colspan="3">No</td><td></td></tr>
<tr><td colspan="3">112</td><td colspan="3">7</td><td colspan="3">143</td><td colspan="3">63</td><td><0.0001</td></tr>
<tr><td colspan="13">No. of ewes in the flock</td></tr>
<tr><td colspan="6">362 ± 25</td><td colspan="6">303 ± 15</td><td>0.031</td></tr>
<tr><td colspan="13">Collaboration with a veterinarian</td></tr>
<tr><td colspan="3">Yes</td><td colspan="3">No</td><td colspan="3">Yes</td><td colspan="3">No</td><td></td></tr>
<tr><td colspan="3">111</td><td colspan="3">8</td><td colspan="3">172</td><td colspan="3">34</td><td>0.011</td></tr>
<tr><td colspan="13">Age of the farmer</td></tr>
<tr><td colspan="3">Up to 50 years</td><td colspan="3">Over 50 years</td><td colspan="3">Up to 50 years</td><td colspan="3">Over 50 years</td><td></td></tr>
<tr><td colspan="3">91</td><td colspan="3">28</td><td colspan="3">106</td><td colspan="3">100</td><td>< 0.0001</td></tr>
<tr><td colspan="13">Length of previous animal farming experience of the farmer</td></tr>
<tr><td colspan="3">≤ 5 years</td><td colspan="3">> 5 years</td><td colspan="3">≤ 5 years</td><td colspan="3">> 5 years</td><td></td></tr>
<tr><td colspan="3">45</td><td colspan="3">74</td><td colspan="3">29</td><td colspan="3">177</td><td>< 0.0001</td></tr>
<tr><td colspan="13">Education of the farmer</td></tr>
<tr><td colspan="2">Primary education</td><td colspan="2">Secondary or post-secondary education</td><td colspan="2">Tertiary education</td><td colspan="2">Primary education</td><td colspan="2">Secondary or post-secondary education</td><td colspan="2">Tertiary education</td><td></td></tr>
<tr><td colspan="2">16</td><td colspan="2">87</td><td colspan="2">16</td><td colspan="2">41</td><td colspan="2">138</td><td colspan="2">27</td><td>0.33</td></tr>
<tr><td colspan="13">Professional involvement in farming</td></tr>
<tr><td colspan="3">Full-time</td><td colspan="3">Part-time</td><td colspan="3">Full-time</td><td colspan="3">Part-time</td><td></td></tr>
<tr><td colspan="3">104</td><td colspan="3">15</td><td colspan="3">188</td><td colspan="3">18</td><td>0.27</td></tr>
</table>

**Daily period at the farm**				
≤ 8 hours	> 8 hours	≤ 8 hours	> 8 hours	
45	74	54	152	0.028
**Family tradition in farming**				
Yes	No	Yes	No	
90	29	193	13	<0.0001
**Presence of working staff in the flock**				
Yes	No	Yes	No	
57	62	66	140	0.005

Table S8. Results of univariable analysis for associations with pregnancy diagnosis by means of ultrasonographic examination in 119 goat herds in Greece.

**Pregnancy diagnosis performed (*n* = 20)**			**Reproductive control not performed (*n* = 99)**			***p***
**Management system applied in the farm**						
Intensive or Semi-intensive	Semi-extensive or Extensive		Intensive or Semi-intensive	Semi-extensive or Extensive		
12	8		26	73		0.003
**Availability of milking parlour**						
Yes	No		Yes	No		
18	2		48	51		0.0007
**No. of does in the flock**						
197 ± 48			246 ± 23			0.038
**Collaboration with a veterinarian**						
Yes	No		Yes	No		
19	1		82	17		0.08
**Age of the farmer**						
Up to 50 years	Over 50 years		Up to 50 years	Over 50 years		
15	5		58	41		0.17
**Length of previous animal farming experience of the farmer**						
≤ 5 years	> 5 years		≤ 5 years	> 5 years		
7	13		17	82		0.07
**Education of the farmer**						
Primary education	Secondary or post-secondary education	Tertiary education	Primary education	Secondary or post-secondary education	Tertiary education	
1	17	2	19	72	8	0.30
**Professional involvement in farming**						
Full-time	Part-time		Full-time	Part-time		
19	1		86	13		0.30

**Daily period at the farm**				
≤ 8 hours	> 8 hours	≤ 8 hours	> 8 hours	
6	14	22	77	0.45
**Family tradition in farming**				
Yes	No	Yes	No	
15	5	88	11	0.10
**Presence of working staff in the flock**				
Yes	No	Yes	No	
11	9	23	76	0.004

Table S9. Associations of the modification of the nutritional regime during pregnancy and the grouping of animals at the end of gestation according to the projected dates of parturition with application of reproductive control in 325 sheep flocks and 119 goat herds in Greece.

Management system	Sheep		Goats	
	**Application of reproductive control**	**No application of reproductive control**	**Application of reproductive control**	**No application of reproductive control**
Modification of the nutritional regime during pregnancy	88 / 108 (81.5%)	141 / 217 (65.0%)	12 / 20 (60.0%)	56 / 99 (56.6%)
No modification of the nutritional regime during pregnancy	20 / 108 (18.5%)	76 / 217 (35.0%)	8 / 20 (40.0%)	43 / 99 (43.4%)
p	0.002		0.78	
Grouping of animals at the end of gestation according to the projected dates of parturition	90 / 108 (83.3%)	124 / 217 (57.1%)	16 / 20 (80.0%)	53 / 99 (53.5%)
No grouping of animals at the end of gestation according to the projected dates of parturition	18 / 108 (16.7%)	93 / 217 (42.9%)	4 / 20 (20.0%)	46 / 99 (46.5%)
p	< 0.0001		0.029	

Table S10. Associations of the modification of the nutritional regime during pregnancy and the grouping of animals at the end of gestation according to the projected dates of parturition with pregnancy diagnosis by means of ultrasonographic examination in 325 sheep flocks and 119 goat herds in Greece.

	Sheep		Goats	
**Management system**	**Pregnancy diagnosis by means of ultrasonographic examination**	**No pregnancy diagnosis by means of ultrasonographic examination**	**Pregnancy diagnosis by means of ultrasonographic examination**	**No pregnancy diagnosis by means of ultrasonographic examination**
Modification of the nutritional regime during pregnancy	106 / 119 (89.1%)	123 / 206 (59.7%)	18 / 20 (90.0%)	50 / 99 (50.5%)
No modification of the nutritional regime during pregnancy	13 / 119 (10.9%)	83 / 206 (40.3%)	2 / 20 (4.0%)	49 / 99 (49.5%)
p	< 0.0001		0.001	
Grouping of animals at the end of gestation according to the projected dates of parturition	100 / 119 (84.0%)	114 / 206 (55.3%)	17 / 20 (85.0%)	52 / 99 (52.5%)
No grouping of animals at the end of gestation according to the projected dates of parturition	19 / 119 (16.0%)	92 / 206 (44.7%)	3 / 20 (15.0%)	47 / 99 (47.5%)
p	< 0.0001		0.007	

Table S11. Results of univariable analysis for associations with number of lambs born per ewe in 325 sheep flocks in Greece.

Lambs born per ewe below national average (1.33) (n = 189)			Lambs born per ewe above national average (1.33) (n = 136)			p
**Location of the farm**						
Northern part of Greece	Central part of Greece	Southern part of Greece	Northern part of Greece	Central part of Greece	Southern part of Greece	
71	94	24	82	43	11	0.0003
**Management system applied in the farm**						
Intensive or Semi-intensive	Semi-extensive or Extensive		Intensive or Semi-intensive	Semi-extensive or Extensive		
98	91		86	50		0.041
**Ewe : ram ratio in the farm**						
1:25.5 (1:17.3)			1:24.1 (1:11.0)			0.37
**Average age of culling ewes (years)**						
5.9 ± 0.1			5.8 ± 0.1			0.78
**Breed of animals**						
Crossbreeds	Imported breeds	Local breeds	Crossbreeds	Imported breeds	Local breeds	
31	69	89	12	70	54	0.014
**Collaboration with a veterinarian**						
Yes	No		Yes	No		
160	29		123	13		0.13
**Application of reproductive control**						
Yes	No		Yes	No		
57	132		51	85		0.17
**Start of mating season in adult animals**						
May (February – October)			May (February – December)			0.37
**Duration of mating season in adult animals (months)**						
3.3 ± 0.2			3.4 ± 0.3			0.61

**Age of the farmer**						
Up to 50 years	Over 50 years		Up to 50 years	Over 50 years		
109	80		88	48		> 0.20
**Length of previous animal farming experience of the farmer**						
≤ 5 years	> 5 years		≤ 5 years	> 5 years		
35	154		39	97		0.031
**Education of the farmer**						
Primary education	Secondary or post-secondary education	Tertiary education	Primary education	Secondary or post-secondary education	Tertiary education	
34	132	23	23	92	20	0.78
**Professional involvement in farming**						
Full-time	Part-time		Full-time	Part-time		
172	17		120	16		0.41
**Daily period at the farm**						
≤ 8 hours	> 8 hours		≤ 8 hours	> 8 hours		
44	145		55	81		0.0009
**Family tradition in farming**						
Yes	No		Yes	No		
169	20		114	22		0.14
**Presence of working staff in the flock**						
Yes	No		Yes	No		
64	125		59	77		0.08

Table S12. Results of univariable analysis for associations with number of kids born per female goat in 119 goat herds in Greece.

**Kids born per female goat below national average (1.30) (*n* = 74)**			**Kids born per female goat above national average (1.30) (*n* = 45)**			***p***
			**Location of the farm**			
Northern part of Greece	Central part of Greece	Southern part of Greece	Northern part of Greece	Central part of Greece	Southern part of Greece	
27	34	13	25	14	6	0.12
			**Management system applied in the farm**			
Intensive or Semi-intensive	Semi-extensive or Extensive		Intensive or Semi-intensive	Semi-extensive or Extensive		
20	54		18	27		0.14
			**Female goat : buck ratio in the farm**			
	26.3 (1:13.9)			18.8 (1:11)		0.45
			**Average age of culling female goats (years)**			
	7.1 ± 0.2			6.7 ± 0.3		0.32
			**Breed of animals**			
Crossbreeds	Imported breeds	Local breeds	Crossbreeds	Imported breeds	Local breeds	
9	23	42	9	22	14	0.025
			**Collaboration with a veterinarian**			
Yes	No		Yes	No		
62	12		39	6		0.67
Yes	No		Yes	No		
6	68		14	31		0.001
			**Start of mating season in adult animals**			
	June (January – December)			June (February – October)		0.86
			**Duration of mating season in adult animals (months)**			
	2.6 ± 0.3			3.1 ± 0.4		0.40

**Age of the farmer**						
Up to 50 years	Over 50 years		Up to 50 years	Over 50 years		
42	32		31	14		0.19
**Length of previous animal farming experience of the farmer**						
≤ 5 years	> 5 years		≤ 5 years	> 5 years		
11	63		13	32		0.06
**Education of the farmer**						
Primary education	Secondary or post-secondary education	Tertiary education	Primary education	Secondary or post-secondary education	Tertiary education	
12	57	5	8	32	5	0.67
**Professional involvement in farming**						
Full-time	Part-time		Full-time	Part-time		
66	8		39	6		0.68
**Daily period at the farm**						
≤ 8 hours	> 8 hours		≤ 8 hours	> 8 hours		
15	59		13	32		0.28
**Family tradition in farming**						
Yes	No		Yes	No		
65	9		38	7		0.60
**Presence of working staff in the herd**						
Yes	No		Yes	No		
18	56		16	29		0.19

Table S13. Number of lambs / kids born per ewe / female goat in accord with breeds of animals in sheep and goat farms in Greece.

Sheep breed [1]	Mean number of lambs born per ewe found in the study	Average number of lambs born per ewe in Greece, as reported in the literature [Zygogiannis 2014 [2]]
Assaf	1.32 ± 0.03	not available
Chios	1.39 ± 0.03	1.75
Crossbreeds	1.26 ± 0.02	not available
Friesarta	1.41 ± 0.07	1.50
Friesian	1.37 ± 0.05	1.80-2.00
Karagouniko	1.46 ± 0.09	1.20-1.40
Lacaune	1.39 ± 0.02	1.63
Local breeds	1.25 ± 0.02	1.05-1.40
Mytilini	1.20 ± 0.01	1.10
Sfakia	1.02 ± 0.07	1.10
p	< 0.0001	
**Goat breed [1]**	**Mean number of kids born per female goat found in the study**	**Average number of kids born per female goat in Greece, as reported in the literature [Zygogiannis and Katsaounis 2009 [3]]**
Alpine	1.38 ± 0.12	1.80-2.00
Crossbreeds	1.34 ± 0.05	not available
Damascus	1.31 ± 0.05	1.70-1.80
Local	1.25 ± 0.02	1.10-1.20
Murcia	1.34 ± 0.05	not available
Saanen	1.35 ± 0.10	1.80-2.00
Skopelos	1.18 ± 0.04	1.34
p	0.40	

[1] Only breeds seen in at least five farms are included.

[2] Zygoyiannis, D.G. *Sheep Production*. Synchroni Paideia, Thessaloniki, 2014.

[3] Zygoyiannis, D.G.; Katsaounis, N.K. *Goat Production*. Synchroni Paideia, Thessaloniki, 2009

Table S14. Age of lambs / kids taken away from dam in accord with management system in sheep and goat farms in Greece.

Management system	Sheep flocks	Goat herds	*p*
Intensive or semi-intensive	44 ± 1 days	58 ± 5 days	0.005
Extensive or semi-extensive	58 ± 2 days	68 ± 4 days	0.0001
p	< 0.0001	0.11	

Table S15. Age of replacement of adult animals in accord with management system in sheep and goat farms in Greece.

Sheep flocks			
**Management system**	**Ewes**	**Rams**	***p***
Intensive or semi-intensive	5.7 ± 0.1 years	4.0 ± 0.1 years	< 0.0001
Extensive or semi-extensive	6.1 ± 0.1 years	4.7 ± 0.2 years	< 0.0001
p	0.005	0.002	
**Goat herds**			
**Management system**	**Female goats**	**Bucks**	***p***
Intensive or semi-intensive	6.3 ± 0.1 years	4.0 ± 0.1 years	< 0.0001
Extensive or semi-extensive	7.2 ± 0.2 years	5.2 ± 0.2 days	< 0.0001
p	0.008	0.002	

Table S16. Associations between the start of the mating period and production parametres assessed in sheep and goat farms in Greece.

Sheep flocks			
**Parametres**	**Start of mating period up to April**	**Start of mating period from May**	***p***
Annual milk production per ewe (L)	223 ± 9	201 ± 6	0.06
Average number of lambs born per ewe	1.36 ± 0.02	1.31 ± 0.01	0.08
Somatic cell counts in bulk-tank milk (cells mL^{-1})	0.508 × 10^6 (0.433 × 10^6 – 0.596 × 10^6)	0.467 × 10^6 (0.424 × 10^6 – 0.515 × 10^6)	0.40
Total bacterial counts in bulk-tank milk (c.f.u. mL^{-1})	423 × 10^3 (302 × 10^3 – 603 × 10^3)	402 × 10^3 (316 × 10^3 – 501 × 10^3)	0.46
Fat content in bulk-tank milk (%)	6.32 ± 0.08	6.15 ± 0.06	0.81
Protein content in bulk-tank milk (%)	4.43 ± 0.03	4.42 ± 0.02	0.68

Goat herds			
**Parametres**	**Start of mating period up to May**	**Start of mating period from June**	***p***
Annual milk production per ewe (L)	189 ± 16	210 ± 16	0.36
Average number of kids born per female goat	1.30 ± 0.03	1.30 ± 0.03	0.99
Somatic cell counts in bulk-tank milk (cells mL^{-1})	0.872 × 10^6 (0.733 × 10^6 – 1.037 × 10^6)	0.814 × 10^6 (0.713 × 10^6 – 0.928 × 10^6)	0.53
Total bacterial counts in bulk-tank milk (c.f.u. mL^{-1})	672 × 10^3 (447 × 10^3 – 1023 × 10^3)	531 × 10^3 (372 × 10^3 – 776 × 10^3)	0.41
Fat content in bulk-tank milk (%)	4.96 ± 0.20	4.67 ± 0.14	0.21
Protein content in bulk-tank milk (%)	3.32 ± 0.11	3.25 ± 0.04	0.51

Table S17. Associations between the application of reproductive control and production parametres assessed in sheep and goat farms in Greece.

**Sheep flocks**			
**Parametres**	**No application of reproductive control**	**Application of reproductive control**	***p***
Annual milk production per ewe (L)	197 ± 6	229 ± 8	0.002
Average number of lambs born per ewe	1.30 ± 0.01	1.37 ± 0.02	0.002
Somatic cell counts in bulk-tank milk (cells mL^{-1})	0.504×10^6 (0.458×10^6 – 0.556×10^6)	0.448×10^6 (0.393×10^6 – 0.511×10^6)	0.19
Total bacterial counts in bulk-tank milk (c.f.u. mL^{-1})	432×10^3 (347×10^3 – 550×10^3)	337×10^3 (251×10^3 – 457×10^3)	0.20
Fat content in bulk-tank milk (%)	6.20 ± 0.06	6.10 ± 0.08	0.32
Protein content in bulk-tank milk (%)	4.45 ± 0.03	4.41 ± 0.02	0.16
**Goat herds**			
**Parametres**	**No application of reproductive control**	**No application of reproductive control**	***p***
Annual milk production per ewe (L)	194 ± 12	235 ± 24	0.07
Average number of kids born per female goat	1.26 ± 0.02	1.45 ± 0.06	0.0002
Somatic cell counts in bulk-tank milk (cells mL^{-1})	0.842×10^6 (0.759×10^6 – 0.935×10^6)	0.808×10^6 (0.596×10^6 – 1.096×10^6)	0.75
Total bacterial counts in bulk-tank milk (c.f.u. mL^{-1})	573×10^3 (427×10^3 – 776×10^3)	727×10^3 (417×10^3 – 1259×10^3)	0.51
Fat content in bulk-tank milk (%)	4.80 ± 0.12	4.63 ± 0.25	0.57
Protein content in bulk-tank milk (%)	3.29 ± 0.06	3.20 ± 0.09	0.50

Table S18. Associations between pregnancy diagnosis by means of ultrasonographic examination and production parametres assessed in sheep and goat farms in Greece.

**Sheep flocks**			
**Parametres**	**No application of reproductive control**	**Application of reproductive control**	***p***
Annual milk production per ewe (L)	186 ± 6	244 ± 8	< 0.0001
Average number of lambs born per ewe	1.29 ± 0.01	1.39 ± 0.01	< 0.0001
Somatic cell counts in bulk-tank milk (cells mL^{-1})	0.501×10^6 ($0.451 \times 10^6 - 0.556 \times 10^6$)	0.458×10^6 ($0.407 \times 10^6 - 0.515 \times 10^6$)	0.19
Total bacterial counts in bulk-tank milk (c.f.u. mL^{-1})	437×10^3 ($347 \times 10^3 - 550 \times 10^3$)	331×10^3 ($251 \times 10^3 - 437 \times 10^3$)	0.20
Fat content in bulk-tank milk (%)	6.14 ± 0.06	6.21 ± 0.07	0.46
Protein content in bulk-tank milk (%)	4.39 ± 0.02	4.48 ± 0.02	0.004
**Goat herds**			
**Parametres**	**No application of reproductive control**	**No application of reproductive control**	***p***
Annual milk production per ewe (L)	190 ± 12	255 ± 26	0.024
Average number of kids born per female goat	1.28 ± 0.02	1.39 ± 0.05	0.027
Somatic cell counts in bulk-tank milk (cells mL^{-1})	0.854×10^6 ($0.764 \times 10^6 - 0.954 \times 10^6$)	0.795×10^6 ($0.587 \times 10^6 - 1.023 \times 10^6$)	0.75
Total bacterial counts in bulk-tank milk (c.f.u. mL^{-1})	589×10^3 ($447 \times 10^3 - 813 \times 10^3$)	589×10^3 ($316 \times 10^3 - 1096 \times 10^3$)	0.51
Fat content in bulk-tank milk (%)	4.81 ± 0.12	4.59 ± 0.29	0.47
Protein content in bulk-tank milk (%)	3.28 ± 0.06	3.24 ± 0.05	0.80

Table S19. Correlations between the age that newborns were taken away from their dams and production parametres assessed in sheep and goat farms in Greece.

Sheep flocks		
**Parametres**	**Correlation coefficient between age and respective parametre**	***p***
Annual milk production per ewe (L)	–0.350	< 0.0001
Somatic cell counts in bulk-tank milk (cells mL^{-1})	0.106	0.028
Total bacterial counts in bulk-tank milk (c.f.u. mL^{-1})	0.062	0.13
Fat content in bulk-tank milk (%)	0.094	0.045
Protein content in bulk-tank milk (%)	–0.049	0.19
**Goat herds**		
**Parametres**	**Correlation coefficient between age and respective parametre**	***p***
Annual milk production per ewe (L)	–0.164	0.038
Somatic cell counts in bulk-tank milk (cells mL^{-1})	0.124	0.09
Total bacterial counts in bulk-tank milk (c.f.u. mL^{-1})	0.012	0.45
Fat content in bulk-tank milk (%)	0.286	0.0008
Protein content in bulk-tank milk (%)	–0.024	0.40

Table S20. Correlations between the age that adult animals were removed from the farm and production parametres assessed in sheep and goat farms in Greece.

Sheep flocks		
**Parametres**	**Correlation coefficient between age and respective parametre**	***p***
Annual milk production per ewe (L)	–0.255	< 0.0001
Average number of lambs born per ewe	–0.062	0.13
Somatic cell counts in bulk-tank milk (cells mL^{-1})	0.076	0.09
Total bacterial counts in bulk-tank milk (c.f.u. mL^{-1})	0.048	0.19
Fat content in bulk-tank milk (%)	0.027	0.31
Protein content in bulk-tank milk (%)	–0.009	0.44
**Goat herds**		
**Parametres**	**Correlation coefficient between age and respective parametre**	***p***
Annual milk production per ewe (L)	–0.234	0.006
Average number of kids born per female goat	–0.169	0.034
Somatic cell counts in bulk-tank milk (cells mL^{-1})	–0.039	0.34
Total bacterial counts in bulk-tank milk (c.f.u. mL^{-1})	–0.121	0.10
Fat content in bulk-tank milk (%)	0.060	0.26
Protein content in bulk-tank milk (%)	–0.035	0.35

Table S21. Associations between criteria evaluated by farmers for sourcing replacement animals from their own farms and production parametres assessed in sheep and goat farms in Greece.

**Sheep flocks**				
**Parametres**	Dam milk production	General animal morphology	Milkability	***p***
Annual milk production per ewe (L)	206 ± 6	207 ± 11	220 ± 12	0.54
Somatic cell counts in bulk-tank milk (cells mL^{-1})	0.476×10^6 (0.451×10^6 – 0.522×10^6)	0.463×10^6 (0.382×10^6 – 0.563×10^6)	0.926×10^6 (0.461×10^6 – 0.652×10^6)	0.33
**Goat herds**				
**Parametres**	Dam milk production	General animal morphology	Milkability	***p***
Annual milk production per ewe (L)	187 ± 12	211 ± 25	266 ± 34	0.030
Somatic cell counts in bulk-tank milk (cells mL^{-1})	0.812×10^6 (0.728×10^6 – 0.909×10^6)	0.463×10^6 (0.675×10^6 – 0.974×10^6)	0.549×10^6 (0.708×10^6 – 1.208×10^6)	0.47
